# A case of infection with a candidate new subtype, tentatively termed M, of human immunodeficiency virus type 1 of group M

**DOI:** 10.1016/j.nmni.2026.101863

**Published:** 2026-10-09

**Authors:** Philippe Colson, Céline Boschi, Marielle Bedotto, Anne Motte, Caroline Solas, Marine Lefebvre, Matthieu Million, Christelle Tomei, Jeremy Delerce, Jacques Fantini

**Affiliations:** a IHU Méditerranée Infection, 19-21 boulevard Jean Moulin, Marseille, 13005, France; b Assistance Publique-Hôpitaux de Marseille (AP-HM), Laboratoire des Agents Infectieux, 19-21 boulevard Jean Moulin, Marseille, 13005, France; c Aix-Marseille Univ., Microbes Evolution PHylogeny and Infections (MEPHI), UR D-258, 27 boulevard Jean Moulin, Marseille, 13005, France; d Assistance Publique-Hôpitaux de Marseille (AP-HM), Hôpital La Timone, Laboratoire de Pharmacocinétique et Toxicologie, Marseille, France; e Assistance Publique-Hôpitaux de Marseille (AP-HM), Service de Maladies Infectieuses, 19-21 boulevard Jean Moulin, Marseille, 13005, France; f Aix-Marseille Univ., Risques Infectieux Tropicaux Microorganismes ÉmergentS (RITMES), D257_UMR MD4, 27 boulevard Jean Moulin, Marseille, 13005, France

**Keywords:** HIV-1, Subtype, Group M, Genome, Classification, Democratic Republic of the Congo, France, Marseille

## Abstract

**Background:**

HIV-1 emergence is estimated to have occurred c.a. 1920s in the Democratic Republic of the Congo (DRC). Only its group M is pandemic. It currently comprises 10 established subtypes. Here, we describe a near-complete HIV-1 genome that meets the definition of a candidate new subtype.

**Methods:**

In-house-designed or commercial PCR primers were used for amplification of HIV-1 DNA from blood. Sequencing used Sanger and next-generation (Oxford Nanopore, Illumina) technologies. Bioinformatic analyses used Samtools, Minimap2, Viroscore, Bootscan, IQ-TREE, iTOL, MEGA12, and Nextclade. Structural analyses used Mutate, HyperChem, and Molegro Molecular Viewer.

**Results:**

The patient originates from the DRC. CD4^+^ T cell counts fluctuated between 351 and 2394/mm3, and plasma viral load, initially 5.0 log_10_copies/mL, reached undetectability on antiretroviral therapy. The obtained near-full length genome (PZ307881) showed a mean nucleotide divergence of 12.7% (range, 11.9-13.7%) with genomes of the 10 established subtypes, while mean divergence was 12.6% (10.0-13.7%) between these 10 subtypes. Phylogeny displayed a star-like tree topology. No recombination was identified. No natural antiretroviral drug resistance was detected. Reverse transcriptase (RT) structure exhibited slight changes compared to subtype B RT that could alter processivity and may favor increased genetic diversity.

**Conclusions:**

These data support a candidate new HIV-1 group M subtype we tentatively termed M pending official taxonomic classification. They indicate that HIV-1 diversity is still not comprehensively deciphered and are in line with the DRC as an epicenter of the HIV-1/M pandemic. They warrant further studies to establish this provisional candidate new subtype by detecting it in other epidemiologically unlinked individuals, and to assess its epidemiological, genotypic and phenotypic features.

## 1 Introduction

Human immunodeficiency viruses (HIV) currently include HIV types 1 and 2 [1]. According to the current classification [2], [3], they belong to family *Retroviridae*, subfamily *Orthoretrovirinae*, genus *Lentivirus*, and species *Lentivirus humimdef1* and *Lentivirus humimdef2*, respectively. HIV type 2 circulated primarily in West Africa, and various indications suggest that it could be disappearing [4]. The emergence of HIV type 1 (HIV-1) is estimated to have occurred around the 1920s in the Democratic Republic of the Congo (DRC) [5]. HIV type 1 (HIV-1) currently comprises four lineages named M (for major), N (for non-M non-O), O (for outlier), and P (to continue in alphabetical order) [6], [7]. Group M—the most prevalent lineage by far and the only one pandemic—is believed to have originated in Kinshasa, the capital of the DRC. Ten subtypes have been delineated for HIV-1 of group M [8], [9]. They are labeled A-D, F-H, J-L, with also 9 sub-subtypes (A1-8, F1-2). The 10 subtypes are deemed to have diverged from one or a few ancestral HIV-1 strains among humans after a single chimpanzee-to-human transmission, for some of them, around the 1950s [10] (https://hiv.lanl.gov/content/sequence/HelpDocs/subtypes-more.html). Within each subtype, sequences are more similar to each other than to sequences from other subtypes, throughout the genomes, and are phylogenetically-related.

The designation of a new subtype requires the identification of at least three viral strains or complete genomes from distinct, epidemiologically unlinked individuals [6], [7], [10], [11]. Phylogenetically, each subtype should form a distinct monophyletic clade, with approximately equal genetic distances separating it from the other subtypes, resulting in a characteristic star-like phylogeny. HIV-1 group M subtypes were reported to differ by a mean nucleotide divergence of approximately 15% at the genome level, with large variations according to the gene [8], [12], [13]. Finally, HIV-1 genomes classified within a given subtype are non-mosaic, distinguishing them from recombinant genomes.

The most recently recognized HIV-1 group M subtype is L [11]. The first two sequences assigned to this subtype were identified in 1983 and 1990 from the DRC. A third sequence (2001, DRC), was reported in 2021. Here, we describe a HIV-1 genome obtained from a sample collected from a woman originating from the DRC. This genome meets multiple criteria supporting its classification as a candidate new HIV-1 group M subtype we tentatively termed M.

## 2 Materials and methods

### 2.1 Tiled targeted PCR amplification and next-generation sequencing

This study focused on HIV-1 DNA from residues of peripheral blood mononuclear cells from a blood sample collected during summer 2023. HIV-1 DNA was extracted using the EZ2 Virus Mini v2.0 kit on an EZ2 Connect instrument (Qiagen, Courtaboeuf, France). To obtain the complete viral genome, a first approach consisted in using conventional PCR primers designed with the Primer3 website (https://primer3.org/) (See Supplementary Methods; Supplementary Table S1). Also, the DeepChek Whole Genome HIV-1 Genotyping assay (ABL Diagnostics, Luxembourg) was used.

### 2.2 Next-generation sequencing

PCR amplicons were subjected to sequencing using Sanger sequencing, as previously described [14], or the Oxford Nanopore next-generation sequencing (NGS) technology on a GridION instrument with the SQK-LSK 109 reagent and a R9 flow cell (Oxford Nanopore Technologies, Oxford, UK), or the Illumina technology on a MiSeq instrument with the COVIDseq assay (Illumina Inc., San Diego, USA). Post-PCR enrichment with the DeepChek Whole Genome HIV-1 Genotyping assay, NGS used Illumina technology on a MiSeq instrument with the COVIDseq assay (Illumina Inc.).

### 2.3 Genome assembly and analysis including comparative genomics and phylogenetic analyses

NGS read mapping was performed on reference genome sequences representing several HIV-1 group M subtypes: B (GenBank accession no. K03455), A1 (MH705133), and L (MN271384). As no major differences were observed between the reference genomes, K03455 was used as the reference for the initial mapping with BWA-MEM (v0.7.19) (https://github.com/lh3/BWA), using the -B 2 option. A hybrid consensus sequence combining K03455 and the reads from our samples was then generated using samtools consensus (https://github.com/samtools/samtools), with the -T option to retain the reference nucleotide in regions with no read coverage, and was used as the reference for all subsequent mapping steps (see Supplementary Methods). Also, raw reads were assembled with MEGAHIT (https://github.com/voutcn/MEGAHIT) and the obtained scaffolds were mapped against the hybrid consensus sequence.

### 2.4 Genome sequence analyses including comparative genomics and phylogenetic analyses

Comparative genomics used the genome obtained here and genomes of the 10 established subtypes collected from the Los Alamos National Laboratory (lanl) HIV Sequence Database (https://www.hiv.lanl.gov/content/sequence/NEWALIGN/align.html) as well as the top ten non-redundant hits recovered through a similarity search using BLAST against the GenBank NCBI nucleotide sequence database (https://blast.ncbi.nlm.nih.gov/Blast.cgi; https://www.ncbi.nlm.nih.gov/genbank/) (See Supplementary Methods). Genetic distance matrices built with BioEdit (https://thalljiscience.github.io/) were analyzed using Microsoft-365 Excel (https://www.microsoft.com/fr-fr/microsoft-365/excel). Sub-subtypes A1 and F1 genomes were used as representative of subtypes A and F, respectively. Statistical analysis used the Openepi online tool (https://www.openepi.com/Menu/OE_Menu.htm). Phylogenetic reconstruction incorporated the genome obtained here together with representative genomes of the 10 established subtypes and the top ten non-redundant hits recovered through BLAST against GenBank (https://blast.ncbi.nlm.nih.gov/Blast.cgi; https://www.ncbi.nlm.nih.gov/genbank/). It were performed with IQ-TREE 3 (v3.1.2) (https://iqtree.github.io/) and Nextclade (https://clades.nextstrain.org/) [15] and visualized using iTOL (https://itol.embl.de/) or MEGA12 (https://www.megasoftware.net/). Simian HIV genome CPZ.GA.88.GAB1.X52154 was used as an outgroup. Nextclade was used to generate an alternative tree, which incorporated a set of 1020 genomes enabled by lanl (https://www.lanl.gov/) (data updated 2024-03-13) and was visualized using Auspice (https://auspice.us), and to generate protein sequences. Recombinant analysis by bootscanning was conducted using SimPlot (v3.5.1) (https://github.com/Stephane-S/Simplot_PlusPlus) [16] with a sliding window of 200 nucleotides and a step of 20 nucleotides.

Genotypic resistance to antiretroviral drugs was assessed using current algorithms of the French National Agency for AIDS Research–Emergent Infectious Diseases AC43 Resistance group (France) (https://hivfrenchresistance.org/) and of the Stanford university HIV drug resistance database (USA) (https://hivdb.stanford.edu/). HIV-1 tropism prediction used geno2pheno (https://coreceptor.geno2pheno.org/).

### 2.5 Molecular modeling of the HIV-1 reverse transcriptase

Molecular modeling simulations used the 1RTD reference file [17] in all-atom mode with the Swiss PDB “Mutate” tool [18]. Interaction energies were minimized by the Polak-Ribière conjugate gradient algorithm of HyperChem v8.0 [19], [20]. HXB2 strain reverse transcriptase (RT) (AAB50259.1) was used as reference. Visualizations and mutation mapping used the Molegro Molecular Viewer [21].

## 3 Results

### 3.1 Case report

The patient, a woman in her sixties from the DRC, was diagnosed with HIV-1 in 1989, with transfusion in the DRC considered the mode of transmission (See Supplementary Results). At the start of treatment, the CD4^+^ T lymphocyte count was 351/mm^3^ and CD4/CD8 ratio was 0.23. Subsequently, the CD4 count fluctuated between 406/mm^3^ in 1999 and 2394/mm^3^ in 2008, and has remained >1000/mm^3^ since 2007, and >500/mm^3^ overall except in 2000 and at time of treatment initiation. Plasma HIV-1 RNA was 5.03 log_10_copies/mL at treatment initiation then from 2008 onward remained undetectable in case of regular virological monitoring except in 2024 (2.2 log_10_). The HIV-1 Western blot (HIV Blot 2.2, MP Diagnostics, Singapore) performed in 2023 only showed reactivities to envelope and capsid proteins (Supplementary Fig. S1).

### 3.2 Sequence analyses, comparative genomics and mutational pattern

The obtained near-full length consensus genome (GenBank no. PZ307881) was 8950 nucleotides in length, covering 92% of the subtype B reference genome K03455, mostly due to sequencing defects at the 5′ and 3′ ends. NGS read mapping to reference genomes representing different HIV-1 group M subtypes as well as *de novo* assembly did not improve or alter the final consensus genome sequence. The 10 best matches in GenBank exhibited 83.29-83.60% nucleotide similarities (See Supplementary Results). These genomes were collected from patients in the DRC (n = 2), USA (4), Netherlands (1), and in Gabon (1), between 1987 and 2018, and were of subtype D (n = 1), J (1), B (6), or not determined. An alignment with representative genomes of the 10 established subtype was 9427 nucleotide long. The genome obtained here exhibited a mean (± standard deviation) nucleotide divergence of 12.69 ± 0.41%, ranging between 11.87 and 13.28% (5th-95th percentiles: 11.96-13.12%), with reference genomes of the 10 established subtypes, while mean divergence was 12.64 ± 0.63%, ranging between 10.01 and 13.73% (12.03-13.38%), for these 10 subtypes between each other (Fig. 1; Table 1). Therefore, nucleotide divergence was highly similar for the candidate new subtype and for the established subtypes with no significant differences in pairwise comparisons.

**Fig. 1:**
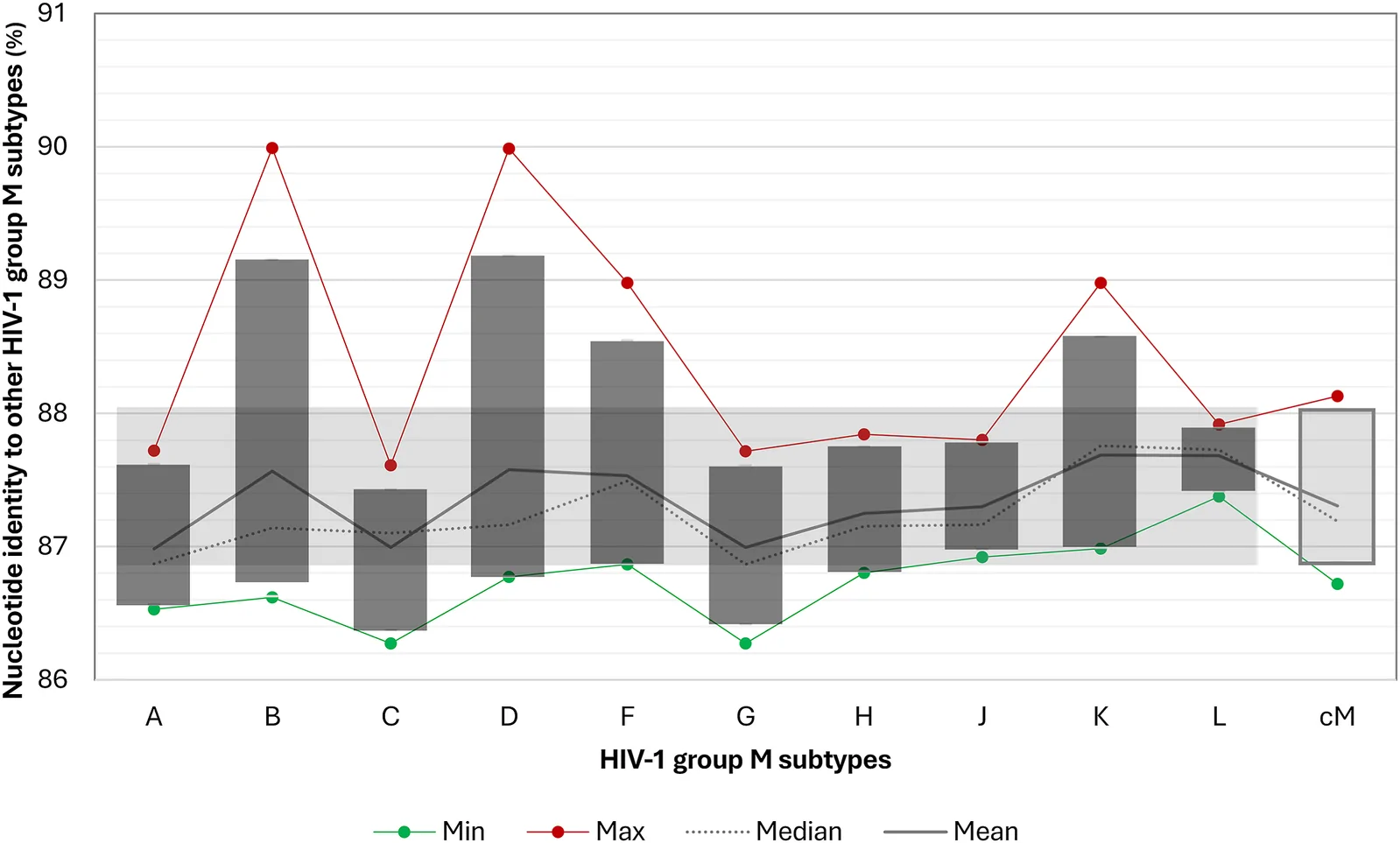
Nucleotide identity to other HIV-1 group M subtypes (%) of established subtypes and of the candidate new subtype M Boxes indicate 0.95 and 0.05 percentiles; cM, Candidate new subtype M.

**Table 1:**
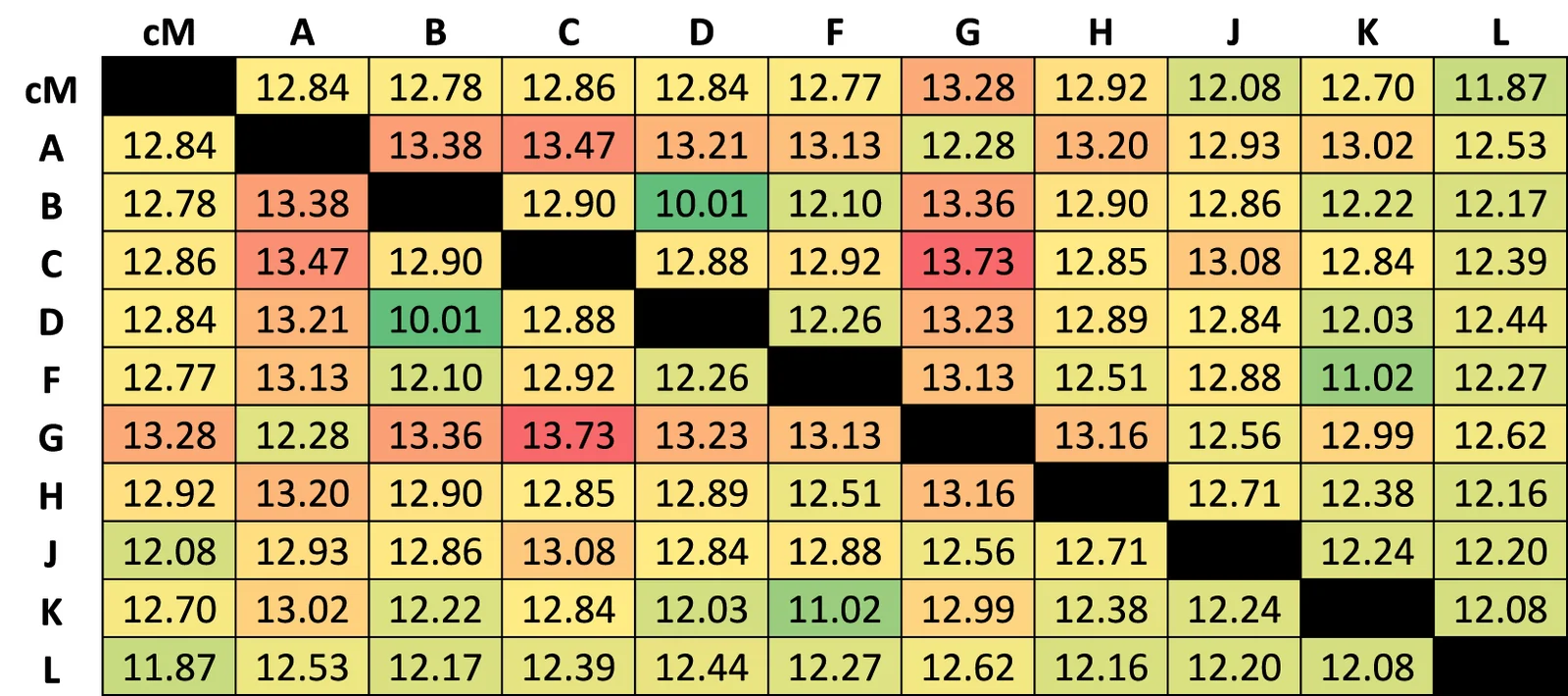
Matrix of nucleotide divergence (%) between established HIV-1 subtypes including the candidate new subtype M.

Nucleotide divergences are indicated in percentages (%). cM, candidate new subtype M.

Similar results were obtained when cutting the genomes into eight fragments with a size ranging between 520 and 2427 nucleotides (mean, 1192 ± 60 nucleotides) corresponding to genome regions determined based on the PCR amplicons or displaying mutational patterns indicating different components of the HIV-1 quasispecies (Supplementary Fig. S2; Supplementary Table S2), and for which mean (± standard deviation) NGS depth was 10,810±7344 reads (range, 562-20,305). Indeed, these genome regions exhibited mean nucleotide divergences ranging between 8.55 and 20.23% (5th-95th percentiles: 11.96-13.12%) for the genome obtained here with reference genomes of the 10 established subtypes, while mean divergences ranged between 8.49 and 20.25% (9.28-19.25%) for these 10 subtypes between each other. Differences between these nucleotide divergences (divergences for the candidate new subtype with the 10 established subtypes minus divergences for the 10 established subtypes between each other) for a given region ranged between −0.07% and +1.03% (Supplementary Table S2).

Phylogenetic analyses showed that the consensus genome sequence of this candidate new subtype formed a distinct branch, as a component of an overall star-like tree (Fig. 2). The closest established HIV-1 subtype was subtype J, with a bootstrap value of 100%. In addition, Nextclade identified the candidate new subtype as an “other clade”, and the visualization by Auspice (https://auspice.us/) of the generated tree that incorporated a set of 1020 genomes enabled by the lanl (data updated 2024-03-13) showed its labelling as belonging to a new node (Supplementary Fig. S3) (See Supplementary Results). Phylogenetic trees built independently from each of the eight genome fragments also showed an overall star-like topology with sequences of the candidate new subtype as a distinct branch (Fig. 3; Supplementary Figs. S4–S9); closest subtypes were B-C-D and F (bootstrap value, 61%), B and D (29%), J (92%), C, A, J and H (79%), J (77%), J and K (83%), G and J (98%), and all subtypes. Finally, bootscan analysis did not identify recombination events for the genome obtained here (Fig. 4). Indeed, the percentage of bootstrap trees almost never exceeded 70-80% and remained <90%, only indicating remote phylogenetic relationships with subtypes D, L, J, A, or H. Taken together, these data indicate that the obtained genome belongs to a candidate new subtype we tentatively termed M as it comes after the last established subtype L [11]. Nonetheless, this report represents a single patient and HIV-1 genome isolate, whereas at least three epidemiologically unlinked genomes are neeed to formally establish a recognized subtype [6].

**Fig. 2:**
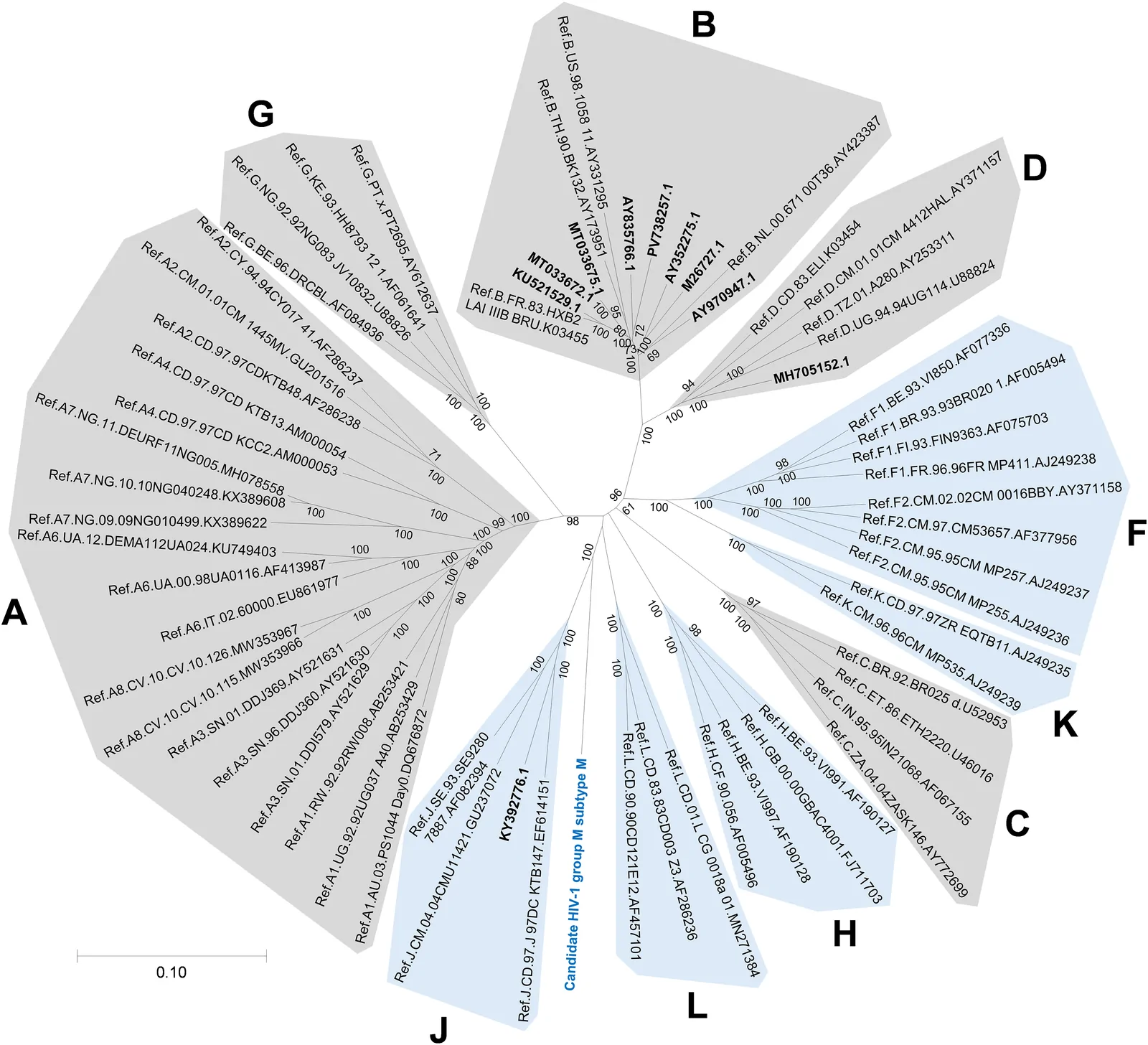
Phylogenetic tree based on complete HIV-1 genomes of the established subtypes and of the candidate new subtype M Each established HIV-1 group M subtype is indicated by a frame with a colored background. Predominant subtypes, grey background, black font; rare subtypes, pale blue background, black font. The candidate new subtype M is indicated by a light blue font. Phylogenetic reconstruction incorporated the genome obtained here together with representative genomes of the 10 established subtypes collected from the Los Alamos National Laboratory HIV Sequence Database (https://www.hiv.lanl.gov/content/sequence/NEWALIGN/align.html) and with the top ten non-redundant hits (indicated by a bold black font) recovered through a similarity search using BLAST (https://blast.ncbi.nlm.nih.gov/Blast.cgi) against the GenBank NCBI nucleotide sequence database (https://www.ncbi.nlm.nih.gov/genbank/). It was performed with IQ-TREE (v3.1.2) (https://iqtree.github.io/) and the tree was visualized using MEGA12 (https://www.megasoftware.net/). The simian HIV genome CPZ.GA.88.GAB1.X52154 was used as an outgroup. Only a subtree is shown here for legilibility.

**Fig. 3:**
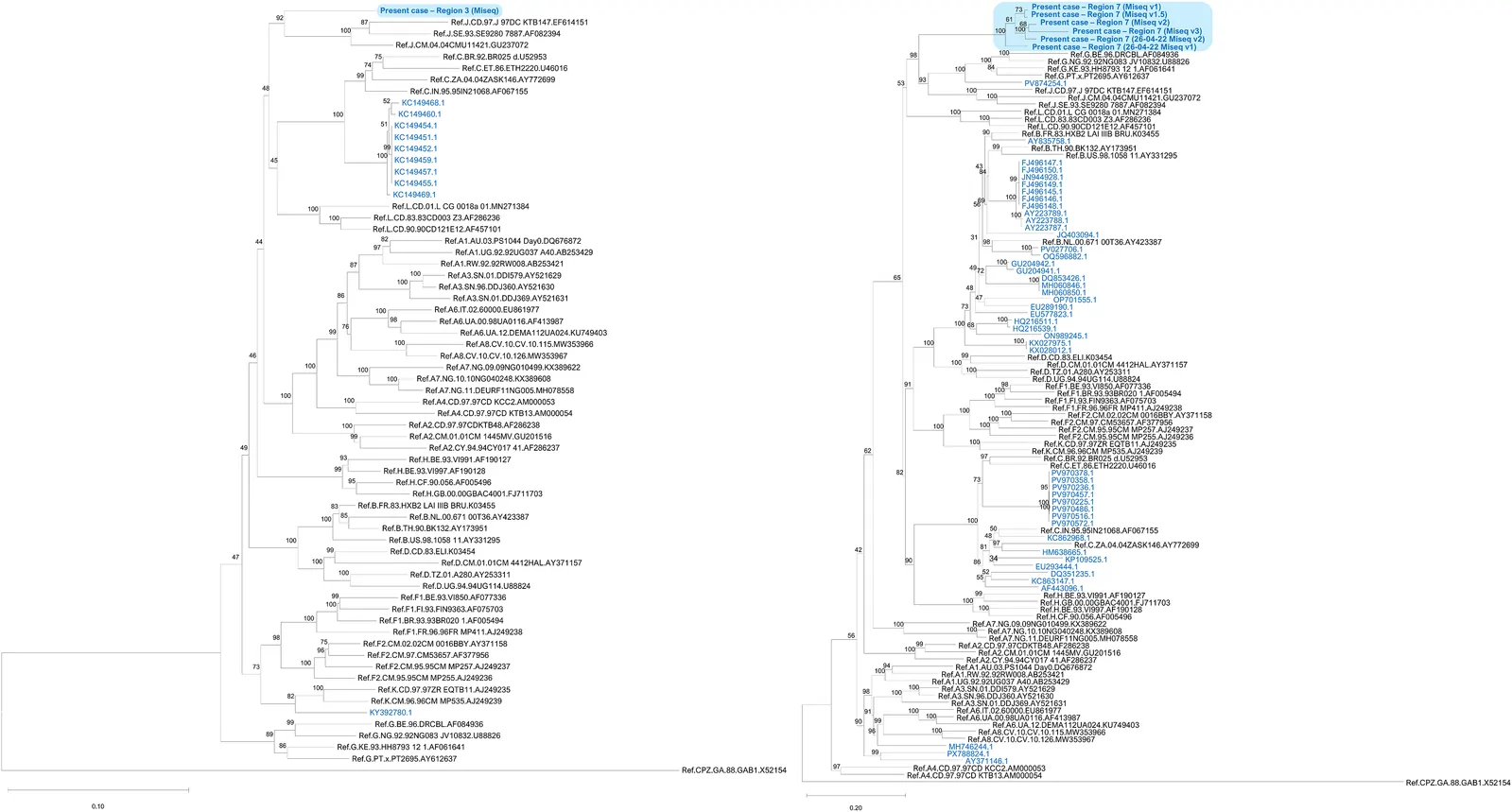
Phylogenetic trees based on two fragments [3], [7] of the HIV-1 genomes Fragments 3 and 7 of the HIV-1 genomes correspond to parts of the *pol* and *env* genes, respectively. Phylogenetic reconstructions incorporated fragments from the genome obtained here (indicated by a blue bold font and a light blue background) together with their top ten non-redundant hits (indicated by a light blue font and their GenBank Accession number) recovered through a similarity search using BLAST (https://blast.ncbi.nlm.nih.gov/Blast.cgi) against the GenBank NCBI nucleotide sequence database (https://www.ncbi.nlm.nih.gov/genbank/), and representative genomes of the 10 established subtypes collected from the Los Alamos National Laboratory HIV Sequence Database (https://www.hiv.lanl.gov/content/sequence/NEWALIGN/align.html) (indicated by a black font). They were performed with IQ-TREE (v3.1.2) (https://iqtree.github.io/) and trees were visualized using MEGA12 (https://www.megasoftware.net/). Simian HIV genome CPZ.GA.88.GAB1.X52154 was used as an outgroup.

**Fig. 4:**
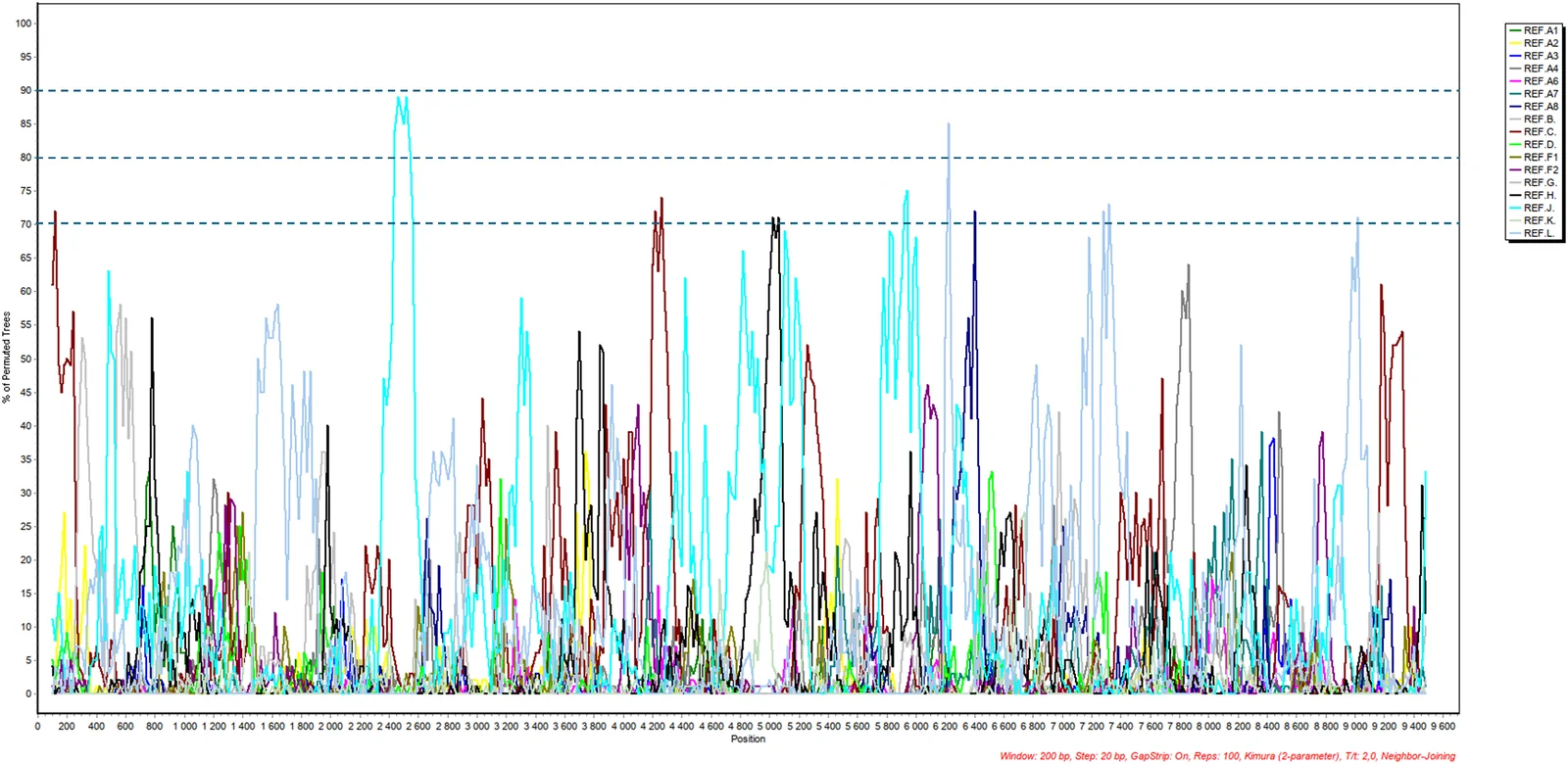
Bootscan analyses for the genomes of the candidate new subtype relatively to those of the established subtypes and sub-subtypes.

No naturally present amino acid mutation was detected that might confer antiretroviral drug resistance to any antiretroviral drug currently available, except one substitution Y181C in RT transiently detected in 2003 during a virological failure episode on nevirapine and one substitution I84V in protease detected in 2007 during virological failure on atazanavir/ritonavir (See Supplementary Results). HIV-1 tropism was predicted to be R5.

Besides, based on available sequences, we observed a mean intra-sample diversity along the HIV-1 genome for this candidate new subtype of 1.61 ± 1.73% (range, 0.00-5.52%), while it was 5.21 ± 1.82% (2.28-10.01%) intra-subtype for the 10 established subtypes. Importantly, all sequences remained distantly related from those of the 10 other established subtypes when considering either nucleotide divergences or phylogeny reconstructions (Fig. 3; Supplementary Figs. S4–S9).

### 3.3 Molecular modeling of the HIV-1 reverse transcriptase

We were interested in RT structure of this new divergent HIV-1 as this is an enzyme central for replication and a target of antiretroviral drugs. The mapping of amino acid differences on this candidate new subtype RT compared to the HIV-1 HXB2 enzyme allowed us to highlight their location on the protein surface (Fig. 5a and b). The 40 mutations were predicted to lead to structural changes in RT loops (Fig. 5c and d), as for the case of the loops formed by amino acids S134-P140 or K281-T296. We observed a closure of the F130–P150 buckle and a general flattening of the K281–P294 loop (Fig. 5e and f). Also, changes in the N57–D76 region, which corresponds to the β3-β4 loop that is functionally important within the RT “fingers” subdomain, are associated with a greater space between RT “fingers” and “palm” [22]. In addition, mutations were predicted to modify the surface electrostatic potential, which nevertheless remains globally electropositive, especially in the ligand interacting area (Fig. 5e and f). Interestingly, we observed that the RT-ligand interaction energy was slightly decreased for the candidate new subtype, from −646 to −512 kJ mol. This is caused by the decrease from 15 to 7 of amino acids involved in ligand interaction (Supplementary Fig. S10).

**Fig. 5:**
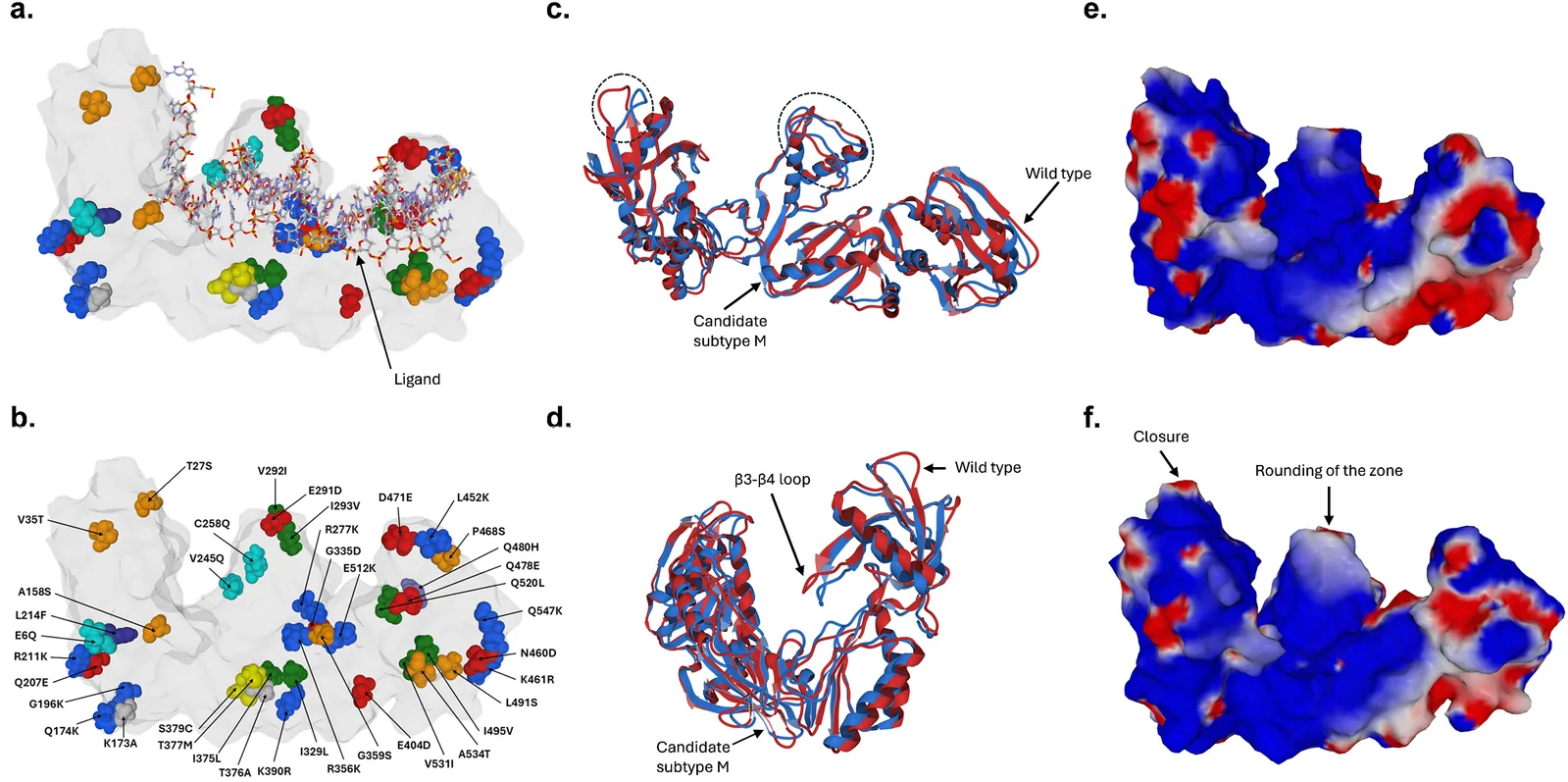
Structural representations of the reverse transcriptase of the candidate new subtype a. Mutations of the candidate new subtype reverse transcriptase (RT) with respect to the reference 1RTD in presence of the ligand; b. Mapping of mutations of the candidate new subtype RT with respect to reference 1RTD. c,d. Two distinct views of RT structure represented in backbones; red color corresponds to the 1RTD file while the blue color corresponds to the candidate new subtype. Examples of loop reshaping are indicated by circles with a black dashed line. e,f. Surface representations of the structure of 1RTD and of the candidate new subtype RT; molecular surface representations are colored according to the electrostatic surface potential: blue, electropositive; red, electronegative; white, neutral.

## 4 Discussion

Present analyses conducted to identify the HIV-1 strain infecting a patient from the DRC monitored at our institute strongly indicate that it belongs to a candidate new subtype within HIV-1 group M. Indeed, the sequences of this candidate new subtype are as divergent from established subtypes as established subtypes are from one another [11], and no recombination events were detected. We have provisionally designated this new candidate subtype as “M,” following the previously established subtype L and the alphabetical order, while noting the potential nomenclatural ambiguity associated with the use of the letter M given that it already designates the major pandemic group of HIV-1.

This candidate new subtype falls among the rarest group M subtypes—alongside subtypes F, H, J, K, and L—each of which accounts for <1% of all global HIV-1 infections [8]. It further points to the DRC as the HIV-1 epidemic epicenter. Rare currently established subtypes are known to circulate primarily in the DRC, and in Angola and Cameroon [8], [23]. Outside sub-Saharan Africa, they are found in Romania, Italy, Spain, Brazil, and Argentina [8]. Thy were initially identified between 1993 (subtype F) and 2002 (L). Heterosexual or vertical transmissions predominated. Genotypic and phenotypic data for these subtypes are limited. No specific characteristics regarding transmissibility, virulence, or pathogenicity have been reported for these rare subtypes. Circulating recombinant forms involving these subtypes have been reported for subtypes F, H, J, and K—but not for subtype L, the one the most recently established. Antiretroviral drug resistance mutations in RT and protease have been reported in subtypes F, H, and J, but not in subtypes K and L.

We observed by NGS a significant diversity among viral sequences obtained from the blood sample from which a genomic sequence was successfully derived. However, each sequence was highly divergent from all others available in GenBank and from reference subtype sequences, regardless the whole genome or individual genomic fragments were considered. In a US study, intra-patient nucleotide diversity of HIV-1 sequences reaching up to 9% was measured in 11.5% of 3303 individuals, suggesting that highly divergent HIV-1 sequences could be detected during prolonged infection in a given individual [24]. Here, intra-host nucleotide divergence ranged from a mean of 1.6% to a maximum of 5.5%, depending on the genomic region. This suggests intra-patient HIV-1 evolution or, possibly, superinfection or coinfection with multiple HIV-1 variants of this candidate new subtype, given the patient's history of stays in the DRC over approximately three decades, although the latter hypothesis appears unlikely given the rarity of this candidate subtype based on current knowledge.

Very limited data are available regarding Western blot profiles in patients infected with rare HIV-1 subtypes. However, it is possible that the absence of antibody reactivities against HIV-1 Pol proteins in the Western blot assay used here may, at least partly, be explained by reduced affinities due to antigenically divergent proteins. Alternatively, and regardless of impaired reactivities due to antigenic diversity, the Western blot profile observed here could be explained by an overall weak antibody response to HIV-1 antigens, which may particularly affect proteins encoded by the pol gene (namely reverse transcriptase, protease, and integrase), as previously reported [25]. Moreover, it has been described that HIV-1 Pol reactivities waned in 17% of 530 patients receiving antiretroviral therapy for at least 12 months [26]. Finally, the Western blot profile observed here may result from a combination of these factors, considering both the antigenic composition of this candidate new subtype and the long-standing nature of this HIV-1 infection that has persisted for more than three decades.

The interpretation of an absence of naturally-occurring resistance mutations for the genome of this candidate new subtype should be considered with caution given the significant difference in genetic background of this genome compared to the genomes of other subtypes including unrare ones. However, this is congruent with similar observations for rare subtypes but also for those the most prevalent worldwide [13]. Based on molecular modeling, we predicted that RT amino acid mutations of this candidate new subtype may cause slight changes in RT loops, including the β3-β4 loop, which is directly involved in enzyme processivity [22], [27]. The conservation of the electrostatic potential throughout the RT ligand interaction zone is consistent with RT essential function. The interaction energy decrease does not compromise the formation of the enzyme-substrate complex but could modulate its stability and consequently RT properties notably processivity [28]. Reduced RT processivity, by promoting dissociation/re-association events of the enzyme-substrate complex, could increase template switching frequency and thus genetic recombinations [29], [30]. However, these hypotheses derive entirely from *in silico* molecular modeling and require *in vitro* biochemical validation.

In summary, present data indicate that HIV-1 diversity is still not comprehensively deciphered, and are in line with previous evidence of the DRC as an epicenter of the HIV-1 group M pandemic and a geographical area of continued circulation of rare HIV-1 subtypes [1], [23], [31]. In addition, they prompt conducting further studies, primarily in the DRC, to obtain additional genomes to attempt establishing this provisional candidate new subtype and its naming by detecting it in at least two more individuals with no direct epidemiological linkage between each other and with the present-case, and to determine its prevalence and potential genotypic (by extended analyses) and phenotypic characteristics.

## CRediT authorship contribution statement

**Philippe Colson:** Writing – review & editing, Writing – original draft, Methodology, Formal analysis, Conceptualization. **Céline Boschi:** Writing – review & editing, Methodology, Formal analysis. **Marielle Bedotto:** Writing – review & editing, Writing – original draft, Methodology, Formal analysis. **Anne Motte:** Writing – review & editing, Formal analysis. **Caroline Solas:** Writing – review & editing, Formal analysis. **Marine Lefebvre:** Writing – review & editing, Writing – original draft, Methodology, Formal analysis. **Matthieu Million:** Writing – review & editing, Formal analysis. **Christelle Tomei:** Writing – review & editing, Formal analysis. **Jeremy Delerce:** Writing – review & editing, Writing – original draft, Methodology, Formal analysis. **Jacques Fantini:** Writing – review & editing, Writing – original draft, Methodology, Formal analysis.

## Data accessibility

The HIV-1 genome obtained here has been deposited in the NBCI GenBank nucleotide sequence database (https://www.ncbi.nlm.nih.gov/genbank/) under accession no. PZ307881.

## Ethics

The present study was registered on the Health Data Access Portal of Marseille Public and University hospitals (Assistance Publique-Hôpitaux de Marseille (AP-HM) and it has been approved by the Ethics and Scientific Committee of AP-HM under No. CSE PADS26-180. Informed consent was obtained from the patient.

## Funding

This work was supported by the French Government under the “Investments for the Future” program managed by the National Agency for Research (ANR), Méditerranée-Infection 10-IAHU-03, by the Contrat Plan Etat-Région, and by the European funding FEDER IHUPERF.

## Declaration of competing interest

The authors declare the following financial interests/personal relationships which may be considered as potential competing interests: PC is a scientific advisor of BioSellal and Triber Global companies. Other authors declare that they have no known competing financial interests or personal relationships that could have appeared to influence the work reported in this paper. Funding sources had no role in the design and conduct of the study; collection, management, analysis, and interpretation of the data; and preparation, review, or approval of the manuscript.
