# Supplementary material for: A case of infection with a candidate new subtype, tentatively termed M, of human immunodeficiency virus type 1 of group M
Source: New Microbes New Infect. 2026 Oct 9;74:101863. doi: 10.1016/j.nmni.2026.101863 (PMC13695309; doi:10.1016/j.nmni.2026.101863)
Supplement: Supplementary file 1 [file mmc1.docx]

**SUPPLEMENTARY MATERIAL**

**for**

**A case of infection with a candidate new subtype, tentatively termed M, of human immunodeficiency virus type 1 of group M**

**Supplementary METHODS**

**Tiled targeted-PCR amplification and next-generation sequencing**

PCR primer design was initially performed using the HIV-1 reference genome HXB2 (GenBank accession number K03455) and was subsequently optimized for selected genomic regions using the HIV-1 group L genome L-CG-0018a-01 (GenBank accession number MN271384). The design parameters selected were a hybridization temperature of 60°C, amplicon sizes ranging from 600 to 1,500 base pairs (bp), and overlaps of 100 to 300 bp between adjacent amplicons. All PCR amplifications were carried out in 25 µL of reaction mixtures using the AmpliTaq Gold 360 Master Mix (Applied Biosystems, Foster City, CA, USA) with primers at a final concentration of 200 nM each and with 5 µL of DNA template. PCR amplification was performed on an ABI 2720 Thermal Cycler (Applied Biosystems) under the following cycling conditions: initial denaturation for 10 min at 95°C, followed by 40 cycles comprising 15 s at 95°C, 45 s at 58°C, and 1 min 30 s at 72°C, with a final extension step for 5 min at 72°C. A second-round PCR was then performed using 2 µL of the first-round cDNA as template, under the same reaction composition and cycling conditions as described above. Amplicons were visualized by electrophoresis on a 1.5% (w/v) agarose gel containing SYBR Safe stain. Also, the DeepChek Assay Whole Genome HIV-1 Genotyping kit (ABL Diagnostics, Luxembourg) was used, according to the manufacturer's recommendations, to attempt amplifying the HIV-1 genome, based on two-round PCR systems that generate five amplicons that overlap to cover the whole viral genome.

PCR amplicons were subjected to sequencing using either Sanger sequencing, as previously described (1), or by the Oxford Nanopore next-generation sequencing (NGS) technology on a GridION instrument with the SQK-LSK 109 reagent and a R9 flow cell (Oxford Nanopore Technologies, Oxford, UK) or the Illumina technology on a MiSeq instrument with the COVIDseq assay (Illumina Inc., San Diego, USA). In case of an enrichment by PCR with the DeepChek Assay Whole Genome HIV-1 Genotyping kit, next-generation sequencing (NGS) used the Illumina technology on a MiSeq instrument with the COVIDseq assay, the paired-end strategy, and a reading of 2x250 bases (Illumina Inc., San Diego, USA).

**Genome assembly and analyses including comparative genomics and phylogenetic analyses**

NGS read mapping was performed against reference genome sequences representing several HIV-1 group M subtypes: subtype B (GenBank accession no. K03455), subtype A1 (MH705133), and subtype L (MN271384). No major differences were observed between the reference genomes; therefore, K03455 was used as the reference for the initial mapping with BWA-MEM (v0.7.19) (https://github.com/lh3/BWA), using the -B 2 option (see Supplementary Methods). A hybrid consensus sequence combining K03455 and the reads from our samples was then generated and used as the reference for all subsequent mapping steps. This hybrid reference was generated using samtools consensus (https://github.com/samtools/samtools), with the -T option to retain the reference nucleotide in regions with no read coverage. In addition, raw NGS reads were assembled with MEGAHIT (https://github.com/voutcn/MEGAHIT) and the obtained scaffolds were mapped against the hybrid reference sequence, but this showed that missing and ambiguous regions were the same between this *de novo* assembly and the direct raw read mapping previously carried out.

For the selection of the PCR amplicon representatives, the reads that mapped outside the expected PCR regions or to regions with a sequencing depth below 100 reads were removed. When known, PCR primers were removed using samtools ampliconclip (v1.23) (https://github.com/samtools/samtools). The reference genome was divided into eight regions, based on our in-house PCR artic-like system or on the presumed PCR regions of the commercial kit (ABL Diagnostics, Luxembourg) also used, and based on major changes observed in the mutation patterns. Amplicon sequences were generated as the consensus sequences of each region using samtools consensus with the -A option. For each NGS run, when multiple amplicons -considering a variant prevalence ≥20%- were detected within the same region, the consensus sequence was duplicated and modified to retain each potential amplicon variant, consisting in the representative amplicons. The Python module pysam (https://github.com/pysam-developers/pysam) was used to identify nucleotides in ambiguous regions within individual reads. A competitive mapping of the reads against the representative amplicons was then performed to ensure that no variant present in more than 80% of the mapped reads was missed, and that all nucleotides corresponding to mutations with a prevalence between 20% and 80% were retained across all representative amplicons. Competitive mapping was iteratively performed until no further updates to the sequences were required. Then, all representative amplicon sequences were mapped against the hybrid reference, and a final consensus sequence was generated using samtools consensus with the following options: -A -a -m simple -d 1. The final consensus sequence was manually curated, as some amplicons displayed apparent deletion–insertion events of equal size, leading to an artificially increased sequence length despite the absence of a true indel. These apparent deletion–insertion regions were therefore manually corrected by replacing the affected nucleotides with the corresponding degenerate nucleotides.

Genome-based phylogenetic analysis incorporated the genome obtained here together with its top ten non-redundant hits recovered through BLAST against GenBank (https://blast.ncbi.nlm.nih.gov/Blast.cgi; https://www.ncbi.nlm.nih.gov/genbank/) and with representative genomes of the 10 established subtypes collected from the Los Alamos National Laboratory HIV Sequence Database (https://www.hiv.lanl.gov/content/sequence/NEWALIGN/align.html). The simian HIV genome CPZ.GA.88.GAB1.X52154 was used as an outgroup. All these sequences were aligned using MAFFT (v7.526) (https://mafft.cbrc.jp/alignment/server/index.html). Phylogeny reconstruction was performed with IQ-TREE 3 (v3.1.2) (https://iqtree.github.io/) with the GTR+F+R5 model and 1,000 ultrafast bootstrap replicates (-B 1000), and trees were visualized using iTOL (https://itol.embl.de/) or MEGA12 (https://www.megasoftware.net/). For phylogenies based on genome regions corresponding to the representative amplicons, the sequences of those latter corresponding to the same genomic region were aligned together with the HIV-1 genome K03455 using MAFFT (v7.526), and phylogenetic trees were generated using IQ-TREE 3 with 1,000 ultrafast bootstrap replicates (-B 1000); selected models were GTR+F+R4, TVM+F+I+G4, GTR+F+R4, TIM2+F+I+G4, GTR+F+I+R3, GTR+F+I+R4, GTR+F+R6 and TVM+F+R5 for genome regions 1, 2, 3, 4, 5, 6, 7 and 8, respectively.

**Supplementary RESULTS**

**Case report**

The patient, a woman in her sixties from the Democratic Republic of Congo (DRC), was diagnosed with HIV-1 in 1989, with transfusion in the DRC considered to be the mode of transmission. Her CDC AIDS stage is B2. She has no co-infection with hepatitis B virus (serological profile of vaccination) or hepatitis C virus (seronegativity). The HIV-1 western blot (HIV Blot 2.2, MP Diagnostics, Singapore) carried out on a serum sample collected in 2023 only showed reactivities to envelope and capsid proteins (Supplementary Figures S1). Her antiretroviral treatments at our institution began in 1998 and first combination therapies comprised didanosine, stavudine, nevirapine, efavirenz, nelfinavir, indinavir, ritonavir, lopinavir, fosamprenavir, and/or atazanavir. Since 2019, this patient has been treated with a combination of antiretrovirals including tenofovir alafenamide, emtricitabine, and bictegravir. At the start of the antiretroviral treatment, the CD4+ T lymphocyte count was 351/mm^3^, with a CD8+ T lymphocyte count of 1,539/mm^3^ and a lymphocyte count of 3,066/mm^3^; the proportion of CD4+ T lymphocytes was 11.4% and the CD4/CD8 ratio was 0.23. Subsequently, the CD4 count fluctuated between 406/mm^3^ in 1999 and 2394/mm^3^ in 2008 with CD4 percentages of 14% and 57%, respectively, and CD4/CD8 ratios of 0.33 and 1.9, respectively. In practice, the CD4 count has remained above 1,000/mm³ since 2007 and has always been above 500/mm³ except for one instance in 2000 and at the time of antiretroviral treatment initiation in 1999. Regarding the plasma HIV-1 RNA load, it was 108,281 copies/mL (5.03 log_10_copies/mL) at the start of antiretroviral therapy; subsequently, there was an alternation between periods of undetectable viral loads and periods of significant viral replication, with viral loads sometimes exceeding 5 log_10_copies/mL. From 2008 onward, the plasma viral load has remained undetectable in the context of regular virological monitoring, with the sole exception of 2024 when a sample showed 162 copies/mL. During these 25 years of follow-up, the patient travelled many times to the DRC, to Kinshasa, with stays of varying lengths.

**Sequence analyses, comparative genomics and mutational pattern**

For the near-full length consensus genome of the candidate new subtype obtained here (GenBank accession no. PZ307881), the 10 best matches (MH705152.1, KY392776.1, AY352275.1, KU521529.1, MT033672.1, AY835766.1, AY970947.1, MT033675.1, M26727.1, PV738253.1) in the NCBI GenBank sequence database (https://www.ncbi.nlm.nih.gov/genbank/) through a similarity search with the BLASTn web application (https://blast.ncbi.nlm.nih.gov/Blast.cgi) were HIV-1 genome exhibiting 83.29%-83.60% nucleotide similarities. Besides, the genome obtained here exhibited a mean (± standard deviation) nucleotide divergence of 12.69±0.41%, ranging between 11.87-13.28% (5^th^-95^th^ percentiles: 11.96-13.12%), with reference genomes of the 10 established subtypes, while mean divergence was 12.64±0.63%, ranging between 10.01-13.73% (12.03-13.38%) for these 10 subtypes between each other (Figure 1; Supplementary Table S2). These levels of nucleotide divergence corresponded to a mean number of 1,197±39 nucleotide differences, ranging between 1,119 and 1,252, for the candidate new subtype genome with reference genomes of the 10 established subtypes, while mean number of differences was 1,192±60, ranging between 944 and 1,294 (5^th^-95^th^ percentiles: 1,134-1,261) for these 10 subtypes between each other.

Based on the genome of this candidate new subtype, no naturally-present amino acid mutation was detected that might confer antiretroviral drug resistance to any antiretroviral drug currently available as far as we can appreciate this genotypically. Thus, no genotypic drug resistance mutations was detected for protease inhibitors, nucleos(t)ide and nonnucleoside reverse transcriptase inhibitors, integrase inhibitors, or lenacapavir (capsid inhibitor). There were two exceptions. A first substitution was Y181C in the reverse transcriptase that was detected in 2003 during an episode of virological failure while the patient was on nevirapine, but that was not detected thereafter. This mutation confers resistance to nevirapine, efavirenz, etravirine and rilpivirine according to both current French National Agency for AIDS Research–Emergent Infectious Diseases AC43 Resistance group (https://hivfrenchresistance.org/) and US Stanford university (https://hivdb.stanford.edu/) algorithms. A second substitution was I84V in the protease that was detected in 2007 also during an episode of virological failure while the patient was on atazanavir/ritonavir. This mutation confers resistance to atazanavir and possible resistance to lopinavir/ritonavir according to the current US Stanford algorithm only. HIV-1 tropism was predicted to be R5 (false positivity rate, 94.1%) according to geno2pheno (https://coreceptor.geno2pheno.org/).

**Supplementary DISCUSSION**

Here, we used both the complete genome and contiguous or slightly overlapping fragments. As with subtype L, we obtained the near-complete genomic sequence for this candidate new subtype by combining metagenomics and next-generation sequencing following an initial sequence enrichment step through PCR amplification of fragments spanning the entire genome with both universal and specific primers.

The decrease in the reverse transcriptase (RT)-ligand interaction energy predicted by molecular modeling for this candidate new subtype can potentially have repercussions on the stability of this complex and therefore, consequently, on the enzymatic properties of the RT of this subtype, in particular its processivity, which is its ability to synthesize a DNA strand continuously on the same template by incorporating a large number of nucleotides before the dissociation of the enzyme-substrate complex (2). Overall, the incomplete conservation of the electrostatic potential and the reduced processivity of the RT may create sequence-context-specific mutational hotspots for this subtype, thereby favoring the differential emergence of certain resistance mutations. However, these findings are supported only by *in silico* molecular modeling and therefore require further validation using *in vitro* biochemical approaches.

**Supplementary REFERENCES**

1.	Tamalet C, Tissot-Dupont H, Motte A, Tourrès C, Dhiver C, Ravaux I, Poizot-Martin I, Dieng T, Tomei C, Bregigeon S, Zaegel-Faucher O, Laroche H, Aherfi S, Mokhtari S, Chaudet H, Ménard A, Brouqui P, Stein A, Colson P. Emergence of uncommon HIV-1 non-B subtypes and circulating recombinant forms and trends in transmission of antiretroviral drug resistance in patients with primary infection during the 2013-2015 period in Marseille, Southeastern France. J Med Virol. 2018 Oct;90(10):1559-1567. doi: 10.1002/jmv.25228. Epub 2018 Jun 11. PMID: 29797570.

2.	Tamalet C, Yahi N, Tourres C, Colson P, Quinson AM, Poizot-Martin I, Dhiver C, Fantini J. Multidrug resistance genotypes (insertions in the beta3-beta4 finger subdomain and MDR mutations) of HIV-1 reverse transcriptase from extensively treated patients: incidence and association with other resistance mutations. Virology. 2000 May 10;270(2):310-6. doi: 10.1006/viro.2000.0261. PMID: 10792990.

3.	Thorvaldsdottir H, Robinson JT, Mesirov JP. Integrative Genomics Viewer (IGV): high-performance genomics data visualization and exploration. Brief Bioinform. 2013 Mar;14(2):178-92. doi: 10.1093/bib/bbs017. Epub 2012 Apr 19. PMID: 22517427; PMCID: PMC3603213.

**Supplementary Figures**

**Supplementary Figures S1. HIV-1 western blot performed on a serum collected in 2023**

The name of HIV-1 proteins corresponding to the observed strips are indicated on the left side of the Figure. Gp160, viral envelope glycoproteins’ precursor; gp120, viral envelope external glycoprotein; p66 and p51, reverse transcriptase: gp41, viral envelope transmembrane glycoprotein; p39, gag precursor/processing product; p31, integrase; p17, matrix.

The HIV-1 western blot was performed using the HIV Blot 2.2 assay (MP Diagnostics, Singapore).

**Supplementary Figures S2. Map of the HIV-1 subtype B strain HXB2 genome and regions 1-8 that enabled reconstructing the genome of the candidate new subtype**

a. The HIV-1 strain HXB2 genome map was adapted from URL: http://hiv-web.lanl.gov/content/hiv-db/COMPENDIUM/2003/0.pdf. b, c. Consensus sequences and reads were visualized by IGV (https://igv.org/) (3). c. S, Sanger technology; M, Illumina technology, MiSeq instrument; N, Oxford Nanopore technology, GridION instrument

**Supplementary Figures S3. Phylogenetic tree based on the HIV-1 genomes generated using Nextclade and Auspice**

The tree was built using Nextclade (https://clades.nextstrain.org/) and visualized using Auspice (https://auspice.us)**.** The figure is a screenshot of the Nextclade tree through Auspice. The HIV-1 genome obtained here is indicated by a star and an arrow.

**Supplementary Figures S4. Phylogenetic tree based on fragment 1 of the HIV-1 genomes**

Phylogenetic reconstruction incorporated a fragment from the genome obtained here (indicated by a blue bold font and a blue background) together with its top ten non-redundant hits (indicated by a light blue font and their GenBank Accession number) recovered through a similarity search using BLAST (https://blast.ncbi.nlm.nih.gov/Blast.cgi) against the GenBank NCBI nucleotide sequence database (https://www.ncbi.nlm.nih.gov/genbank/), and representative genomes of the 10 established subtypes collected from the Los Alamos National Laboratory HIV Sequence Database (https://www.hiv.lanl.gov/content/sequence/NEWALIGN/align.html) (indicated by a black font). It was performed with IQ-TREE (v3.1.2) (https://iqtree.github.io/) and the tree was visualized using MEGA12 (https://www.megasoftware.net/). Simian HIV genome CPZ.GA.88.GAB1.X52154 was used as an outgroup.

**Supplementary Figures S5. Phylogenetic tree based on fragment 2 of the HIV-1 genomes**

Phylogenetic reconstruction incorporated a fragment from the genome obtained here (indicated by a blue bold font and a blue background) together with its top ten non-redundant hits (indicated by a light blue font and their GenBank Accession number) recovered through a similarity search using BLAST (https://blast.ncbi.nlm.nih.gov/Blast.cgi) against the GenBank NCBI nucleotide sequence database (https://www.ncbi.nlm.nih.gov/genbank/), and representative genomes of the 10 established subtypes collected from the Los Alamos National Laboratory HIV Sequence Database (https://www.hiv.lanl.gov/content/sequence/NEWALIGN/align.html) (indicated by a black font). It was performed with IQ-TREE (v3.1.2) (https://iqtree.github.io/) and the tree was visualized using MEGA12 (https://www.megasoftware.net/). Simian HIV genome CPZ.GA.88.GAB1.X52154 was used as an outgroup.

**Supplementary Figures S6. Phylogenetic tree based on fragment 4 of the HIV-1 genomes**

Phylogenetic reconstruction incorporated a fragment from the genome obtained here (indicated by a blue bold font and a blue background) together with its top ten non-redundant hits (indicated by a light blue font and their GenBank Accession number) recovered through a similarity search using BLAST (https://blast.ncbi.nlm.nih.gov/Blast.cgi) against the GenBank NCBI nucleotide sequence database (https://www.ncbi.nlm.nih.gov/genbank/), and representative genomes of the 10 established subtypes collected from the Los Alamos National Laboratory HIV Sequence Database (https://www.hiv.lanl.gov/content/sequence/NEWALIGN/align.html) (indicated by a black font). It was performed with IQ-TREE (v3.1.2) (https://iqtree.github.io/) and the tree was visualized using MEGA12 (https://www.megasoftware.net/). Simian HIV genome CPZ.GA.88.GAB1.X52154 was used as an outgroup.

**Supplementary Figures S7. Phylogenetic tree based on fragment 5 of the HIV-1 genomes**

Phylogenetic reconstruction incorporated a fragment from the genome obtained here (indicated by a blue bold font and a blue background) together with its top ten non-redundant hits (indicated by a light blue font and their GenBank Accession number) recovered through a similarity search using BLAST (https://blast.ncbi.nlm.nih.gov/Blast.cgi) against the GenBank NCBI nucleotide sequence database (https://www.ncbi.nlm.nih.gov/genbank/), and representative genomes of the 10 established subtypes collected from the Los Alamos National Laboratory HIV Sequence Database (https://www.hiv.lanl.gov/content/sequence/NEWALIGN/align.html) (indicated by a black font). It was performed with IQ-TREE (v3.1.2) (https://iqtree.github.io/) and the tree was visualized using MEGA12 (https://www.megasoftware.net/). Simian HIV genome CPZ.GA.88.GAB1.X52154 was used as an outgroup.

**Supplementary Figures S8. Phylogenetic tree based on fragment 6 of the HIV-1 genomes**

Phylogenetic reconstruction incorporated a fragment from the genome obtained here (indicated by a blue bold font and a blue background) together with its top ten non-redundant hits (indicated by a light blue font and their GenBank Accession number) recovered through a similarity search using BLAST (https://blast.ncbi.nlm.nih.gov/Blast.cgi) against the GenBank NCBI nucleotide sequence database (https://www.ncbi.nlm.nih.gov/genbank/), and representative genomes of the 10 established subtypes collected from the Los Alamos National Laboratory HIV Sequence Database (https://www.hiv.lanl.gov/content/sequence/NEWALIGN/align.html) (indicated by a black font). It was performed with IQ-TREE (v3.1.2) (https://iqtree.github.io/) and the tree was visualized using MEGA12 (https://www.megasoftware.net/). Simian HIV genome CPZ.GA.88.GAB1.X52154 was used as an outgroup.

**Supplementary Figures S9. Phylogenetic tree based on fragment 8 of the HIV-1 genomes**

Phylogenetic reconstruction incorporated a fragment from the genome obtained here (indicated by a blue bold font and a blue background) together with its top ten non-redundant hits (indicated by a light blue font and their GenBank Accession number) recovered through a similarity search using BLAST (https://blast.ncbi.nlm.nih.gov/Blast.cgi) against the GenBank NCBI nucleotide sequence database (https://www.ncbi.nlm.nih.gov/genbank/), and representative genomes of the 10 established subtypes collected from the Los Alamos National Laboratory HIV Sequence Database (https://www.hiv.lanl.gov/content/sequence/NEWALIGN/align.html) (indicated by a black font). It was performed with IQ-TREE (v3.1.2) (https://iqtree.github.io/) and the tree was visualized using MEGA12 (https://www.megasoftware.net/). Simian HIV genome CPZ.GA.88.GAB1.X52154 was used as an outgroup.

**Supplementary Figures S10. Energy of interaction between the HIV-1 reverse transcriptases and the nucleic acid substrate**

a, c. 1RTD reverse transcriptase; b, d. Candidate new subtype M reverse transcriptase. Upper panels: amino acids critical for the enzyme-substrate interaction. Lower panels: same views without the substrate

**Supplementary Tables**

**Supplementary Table S1. In-house PCR primers used to amplify genome fragments of the candidate new HIV-1 group M subtype**

| Name of primer | Coordinates in reference to genome GenBank Accession no. | Orientation | Sequence (5'-3') | Primer size (nucleotides) | Amplicon size (nucleotides) | Comment |
| --- | --- | --- | --- | --- | --- | --- |
| 1_HIV-1MM_MBF | 188-860 | Forward | AGAGAACACCAGCTTGTTACACC | 23 | 673 | In-house designed 'artic-like' pan-HIV-1/M system |
| 1_HIV-1MM_MBR |  | Reverse | CCTGGCCTTAACCGAATTTT | 20 |  |  |
| 2_HIV-1MM_MBF | 681-1933 | Forward | GCTCTCTCGACGCAGGACT | 19 | 1253 | In-house designed 'artic-like' system specific of the present genome |
| 2_HIV-1MM_MBR |  | Reverse | AAARTTGCCTCTCTGCATCATT | 22 |  |  |
| 3_HIV-1MM_MBF | 1814-2608 | Forward | CAYTAGAAGAAATGATGACAGCATG | 25 | 795 | In-house designed 'artic-like' pan-HIV-1/M system |
| 3_HIV-1MM_MBR |  | Reverse | TTTGGGCCATCCATTCCT | 18 |  |  |
| 4_HIV-1MM_MBF | 2484-3889 | Forward | GGACCTACACCTGTCAACATAATTG | 25 | 1406 | In-house designed 'artic-like' pan-HIV-1/M system |
| 4_HIV-1MM_MBR |  | Reverse | TTAGCTGCCCCATCTACATAGAA | 23 |  |  |
| 5_HIV-1MM_MBF | 3334- 4371 | Forward | GTCAATGACATACAGAAGTTAGTGGG | 26 | 1038 | Diagnostic-based amended pan-HIV-1/M system |
| 5_HIV-1MM_MBR |  | Reverse | CTTGTCCATGCATGGCTTC | 19 |  |  |
| 6_HIV-1MM_MBF | 3774-5266 | Forward | ACCTGGATTCCTGAGTGGGA | 20 | 1493 | In-house designed 'artic-like' pan-HIV-1/M system |
| 6_HIV-1MM_MBR |  | Reverse | CTCCTGTMTGCAGACCCCAA | 20 |  |  |
| 7_HIV-1MM_MBF | 4371-5097 | Forward | GAAGCCATGCATGGACAAG | 19 | 727 | Diagnostic-based amended pan-HIV-1/M system |
| 7_HIV-1MM_MBR |  | Reverse | TCTAATCCTCATCCTGTCTACTTGC | 25 |  |  |
| 8_HIV-1MM_MBF | 5036-6451 | Forward | GAAAACAGATGGCAGGTGATG | 21 | 1416 | In-house designed 'artic-like' system specific of the present genome |
| 8_HIV-1MM_MBR |  | Reverse | CAGGCATGTGTAGCCCAGA | 19 |  |  |
| 9_HIV-1MM_MBF | 6343-7758 | Forward | TCTATTATGGGGTACCTGTGTGG | 23 | 1416 | In-house designed 'artic-like' system specific of the present genome |
| 9_HIV-1MM_MBR |  | Reverse | CCAAGAACCCGAGAAACAAA | 20 |  |  |
| 10_HIV-1MM_MBF | 7653-9062 | Forward | GTAAAAATTGAACCACTAGGAGTAGCA | 27 | 1410 | In-house designed 'artic-like' system specific of the present genome |
| 10_HIV-1MM_MBR |  | Reverse | TGAATTAGCCCATCCAGTCC | 20 |  |  |
| 11_HIV-1MM_MBF | 8759-9610 | Forward | AAGAATAAGACAGGGCTTGGAAA | 23 | 852 | In-house designed 'artic-like' pan-HIV-1/M system |
| 11_HIV-1MM_MBR |  | Reverse | AGGCTTAAGCAGTGGGTTCC | 20 |  |  |

**Supplementary Table S2. Nucleotide similarities for the genome fragments of the candidate new HIV-1 group M subtype compared to their counterparts in HIV-1 genomes of the 10 established subtypes and for genome fragments of these 10 established subtypes between each other**

| **Genome region** |  | **1** | **2** | **3** | **4** | **5** | **6** | **7** | **8** |
| --- | --- | --- | --- | --- | --- | --- | --- | --- | --- |
| **Region size (nucleotides)** |  | **1125** | **791** | **1401** | **520** | **873** | **1197** | **2427** | **1650** |
| **Nucleotide similarities for the genome fragments of the candidate new subtype vs those of the 10 established subtypes** | Mean | 13,2 | 12,54 | 11,21 | 12,03 | 8,55 | 12,4 | 20,23 | 18,42 |
|  | Standard deviation | 1,12 | 1,53 | 0,54 | 0,83 | 0,54 | 0,89 | 0,54 | 1,15 |
|  | Minimum | 11,47 | 10,62 | 10,28 | 10,96 | 7,79 | 10,19 | 19,78 | 15,64 |
|  | Maximum | 14,4 | 14,92 | 11,85 | 13,27 | 9,39 | 13,37 | 21,26 | 19,45 |
|  | Median | 12,8 | 12,9 | 11,21 | 11,92 | 8,36 | 12,53 | 19,9 | 18,42 |
|  | Percentile 0.05 | 11,82 | 10,72 | 10,36 | 11,04 | 7,93 | 11,03 | 19,81 | 16,65 |
|  | Percentile 0.95 | 14,36 | 14,72 | 11,79 | 13,12 | 9,35 | 13,23 | 21,1 | 19,38 |
| **Nucleotide similarities for the genome fragments of the 10 established subtypes between each other** | Mean | 13,22 | 12,18 | 10,75 | 11,24 | 8,49 | 12,47 | 20,25 | 17,39 |
|  | Standard deviation | 1,52 | 1,51 | 0,9 | 1,14 | 0,62 | 0,98 | 1,23 | 1,54 |
|  | Minimum | 8,36 | 9,23 | 7,92 | 8,08 | 6,87 | 9,52 | 15,99 | 12,91 |
|  | Maximum | 16,36 | 14,92 | 12,13 | 13,27 | 9,97 | 14,37 | 22,21 | 19,45 |
|  | Median | 12,98 | 11,88 | 10,78 | 11,35 | 8,48 | 12,53 | 20,31 | 17,64 |
|  | Percentile 0.05 | 10,76 | 9,86 | 9,42 | 9,23 | 7,56 | 10,61 | 18,58 | 14,67 |
|  | Percentile 0.95 | 15,91 | 14,41 | 11,92 | 12,88 | 9,39 | 13,95 | 21,84 | 19,27 |
